# Primary Neuroendocrine Carcinoma of the Cerebellopontine Angle: A Case Report and Literature Review

**DOI:** 10.7759/cureus.27564

**Published:** 2022-08-01

**Authors:** Enrique Caro-Osorio, Luis A Perez-Ruano, Hector R Martinez, Ana G Rodriguez-Armendariz, Dulce M Lopez-Sotomayor

**Affiliations:** 1 Neurosurgery, Tecnologico de Monterrey, Monterrey, MEX; 2 Neurological Surgery, Tecnologico de Monterrey, Monterrey, MEX; 3 Neurology, Tecnologico de Monterrey, Monterrey, MEX; 4 Pathology and Laboratory Medicine, Tecnologico de Monterrey, Monterrey, MEX

**Keywords:** brain tumor, neuroendocrine carcinoma, brain metastasis, cerebellopontine angle, neuroendocrine neoplasm

## Abstract

Primary intracranial neuroendocrine tumors are extremely rare malignancies with very few cases reported in the world literature. We describe a primary neuroendocrine carcinoma arising from the right cerebellopontine angle, the second case that has been described in this location. The possible origin in this place and treatment are described.

A 29-year-old male patient, diagnosed with schwannoma of the right cerebellopontine angle, and treated with radiosurgery at another institution, came to our hospital six months later, The patient presented with a history of rapid progression of numbness on the right side of the face, diplopia, dizziness, vomiting, and facial palsy. On examination, the right cranial nerves V, VI, VII, VIII, and IX were affected. The MRI showed tumor growth occupying the right cerebellopontine angle, with compression of the brain stem and cerebellum. A right retromastoid craniectomy removed the tumor partially and the histopathological examination revealed a high-grade neuroendocrine carcinoma.

We describe a primary neuroendocrine tumor of the brain that, despite its rarity, must be considered in the differential diagnosis. There are currently no guidelines for the management of these tumors. According to previously reported cases, surgery is the first line of treatment, followed by radiotherapy or chemotherapy. We consider that such a rare case is needed to be reported for a better understanding of the disease and its neurobiology.

## Introduction

Neuroendocrine neoplasms (NENs) are a heterogeneous and uncommon group of malignancies with varied histology. Rarely, these tumors can occur intracranially. There are nine reported cases with a primary origin in the central nervous system (CNS) [1-10]. The cerebellopontine angle (CPA) location has been reported in only one previous case [1]. There are two specific characteristics of these NENs: one is the identification of dense-core granules in electron microscopy, similar to those present in serotonergic neurons, which store monoamines, and the other feature refers to the synthesis and secretion of those monoamines [11]. They are divided into tumors and carcinomas, depending on histologic characteristics, such as the presence of necrosis and mitosis, and the Ki67 proliferation index [11-12].

Because of the wide and even distribution of the endocrine cells, these tumors can arise in almost any site of the body, but the most common origin is in the gastrointestinal tract and bronchi. Brain metastasis has been reported in 1.3-1.4% of the patients [13], but primary NENs are unusual. This report aims to present a case of a neuroendocrine carcinoma (NEC) arising from the CPA, describe the main characteristics of the primary NENs of the CNS that have been reported in the literature, and observations on its origin in this location.

## Case presentation

**Figure 1:**
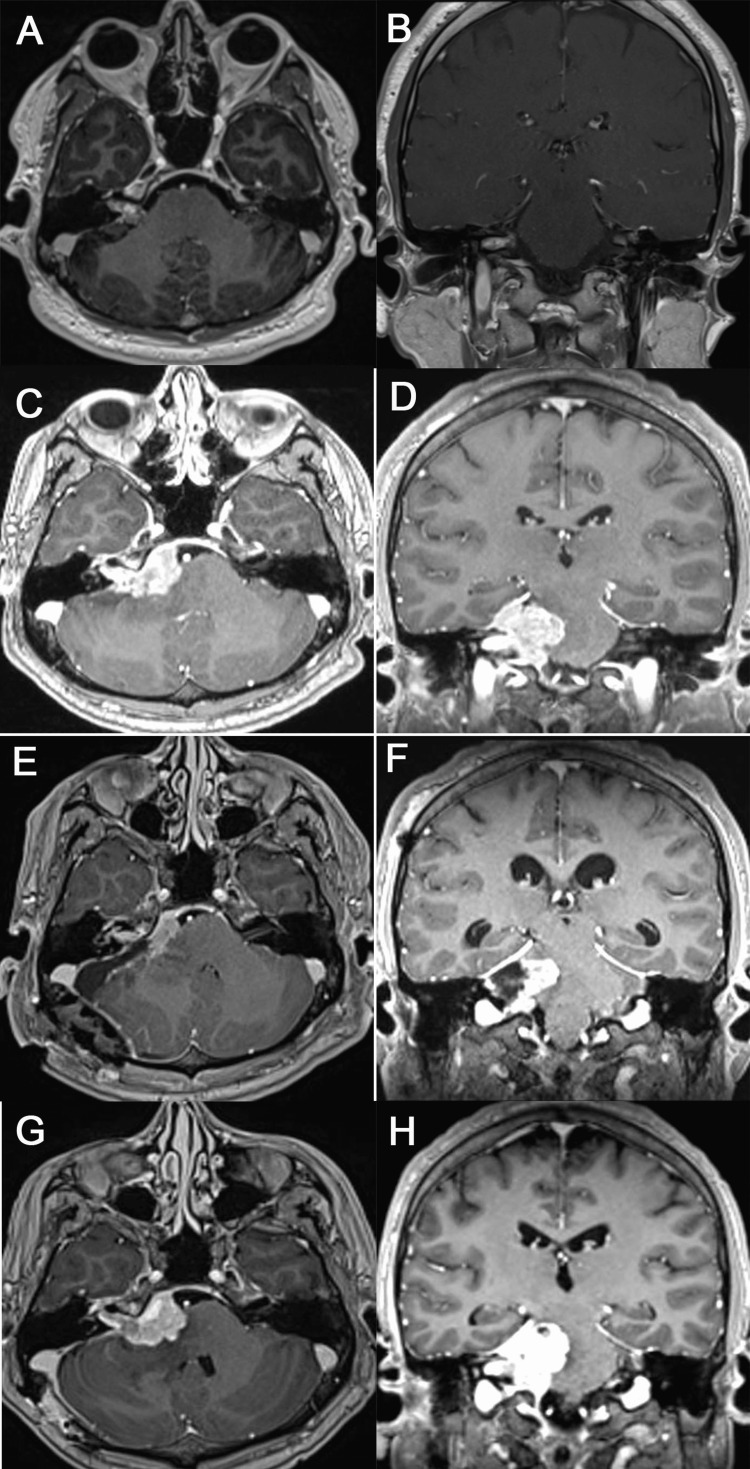
Preoperative and follow-up magnetic resonance imaging Contrast-enhanced T1-weighted axial (A) and coronal (B) demonstrates a mass in the right internal acoustic canal. Six months after radiosurgery treatment contrast-enhanced T1-weighted axial (C) and coronal (D) reveals tumor growth with compression of the brain stem. After the right retromastoid craniectomy approach, contrast-enhanced T1-weighted axial (E) and coronal (F) demonstrated residual tumor. Four cycles after chemotherapy, contrast-enhanced T1-weighted axial (G) and coronal (H) showed tumor progression.

A 29-year-old male, presented at another institution, with progressive right facial palsy, vertigo, nausea, and vomiting. A magnetic resonance image (MRI) demonstrated a right tumor in the internal auditory canal, protruding to the cisterna of the CPA. It was assumed to be a schwannoma of the right vestibulocochlear nerve (Figures 1A-1B). The patient underwent radiosurgery treatment. Six months later, he came to our hospital due to a rapidly progressive numbness on the right side of the face, diplopia, dizziness, nausea, vomiting, and right facial palsy. On admission, the neurologic examination revealed, right peripheral facial palsy with facial anesthesia, right eye in adduction, hearing loss, decreased elevation of the soft palate, and right-side deviation of the tongue on the protrusion. All these signs advocate the involvement of the V, VI, VII, VIII, and IX cranial nerves. There was also scattering speech, gait instability, and right dysmetria suggestive of cerebellar affection.

A head MRI showed tumor growth, occupying the right CPA, with compression of the brain stem and extension to the cerebellomedullary cistern (Figures 1C-1D). The patient underwent a right retro-mastoid craniectomy approach, and an extra-axial vascularized tumor was evidenced, infiltrating the cranial nerves and brain stem, with areas of soft and solid consistency of gray-pink color. Partial tumor resection was performed because dissection of the tumor from the involved lower cranial nerves was complicated by the presence of cardiac asystole, which reversed spontaneously when the tumor was stopped from being manipulated.

**Figure 2:**
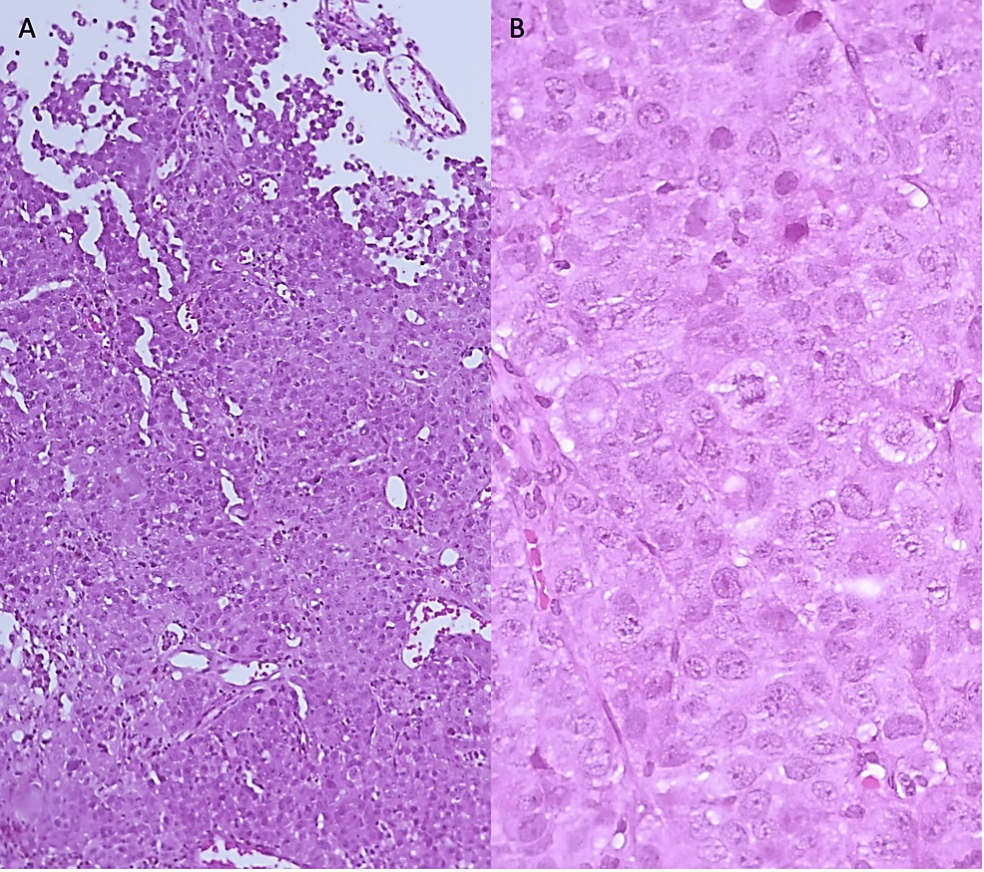
Hematoxylin and eosin stain A) Sheets of cells large cells with big nuclei (H&E, 100x). B) Polyhedral large cells with round nuclei, granular chromatin, and nucleoli. Frequent mitoses were seen (H&E, 400x).

**Figure 3:**
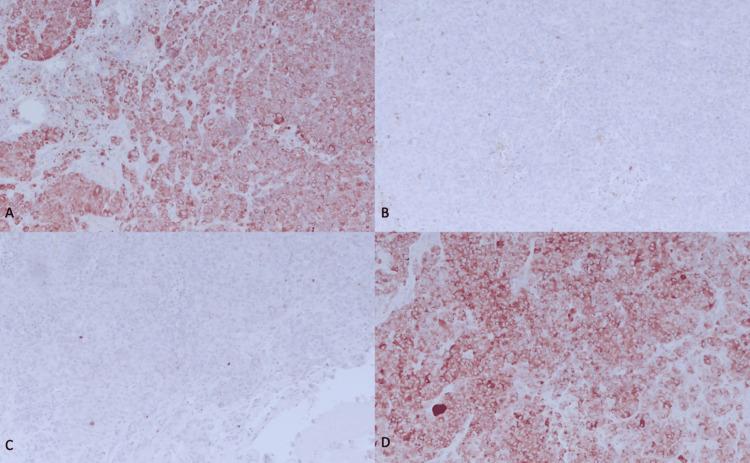
Immunohistochemistry profile A: CK AE1/AE3 positive. B: CD45 negative. C: Melan-A negative. D: Synaptophysin positive.

Histopathological examination revealed a poorly differentiated carcinoma with solid sheets of large, polyhedral cells with amphophilic and ample cytoplasm (Figure 2), with folded nuclei with granular chromatin, some nucleoli, and frequent mitoses (Figure 3). Some cells had brown pigment in their cytoplasm. Immunohistochemistry markers for pan CK, EMA, CK7, chromogranin A, and INSM1 were positive; GFAP, CD45, melan-A, and CK20 were negative; and the proliferation index with Ki67 was 70%. Because of the morphology and immunohistochemistry profile, a large cell neuroendocrine carcinoma was diagnosed.

A thorough evaluation to exclude any evidence of extracranial NENs was performed, including computed tomography (CT) of the thorax, abdomen, and pelvis, which was negative for other tumors. A positron emission tomography-computed tomography (PET-CT) with 18-fluorodeoxyglucose (FDG) was performed to exclude tumor spread that was negative for extracranial NENs. The patient underwent four cycles of chemotherapy (ChT) of cisplatin and etoposide. And trying to strengthen the ChT, a course of monoclonal antibody and an immune-checkpoint inhibitor pembrolizumab was included. Despite this, he experienced worsening of his facial symptoms and numbness of the other side of the face, as well as difficulty swallowing. An MRI eight weeks after surgery revealed huge disease progression and a severe displacement of the brain stem (Figures 1G-1H). Thirteen weeks after surgery, the patient died.

## Discussion

Neuroendocrine tumors (NETs) are relatively uncommon tumors arising from enterochromaffin cells located in many organs such as the gut and lung [14]. They arise most commonly in the gastrointestinal tract (62-67%) or the bronchopulmonary airways (22-27%) [15].

These cells are found diffusely throughout different organs and are well described throughout the gastrointestinal tract, the respiratory tract, and the central nervous system (CNS) [16-17]. Many epithelial cells and their progenitors may be differentiated in neuroendocrine cells and originate NETs in various anatomical locations including the pineal gland [8], sellar and hypothalamus, cavernous sinus, frontal convexity third ventricle, and CPA (Table 1) [1-10]. Neuroendocrine cells are naturally involved in the coordination of neurotransmitter-initiated synthesis and the release of biologically active substances. Therefore, NENs possess unique properties, such as secreting physiologically active amines and peptidyl hormones, which allow NETs to retain unique methods for identification [18].

Intracranial involvement by a NEN is most seen as hematogenous metastasis. Its presentation is common 13 months after the initial diagnosis, on average. The incidence of patients with NENs having brain metastases is <5%. Furthermore, only 1.4% of metastatic brain tumors are NENs, and most of these lesions originate from the lung. There is no gender preference for the occurrence of brain metastases. When patients do present with metastatic NENs to the brain, they typically present with other local and distant metastases [13,19]. Brain metastases are associated with poor prognosis, with a median overall survival of eight months from the diagnosis of CNS involvement [12]. On imaging, there is also no specificity. Bone erosion on CT scans, hypointensity on T1WI (T1 weighted image), hyperintensity on T2WI, and homogeneous enhancement are common characteristics.

Primary intracranial NECs are rarely mentioned in the literature, accounting for only 11 cases until the preparation of this manuscript (Table 1) [1-10]. The first case was described by Porter et al., in 2000 [1]. The presentation of this case was similar to ours in the location (right CPA), but different in histopathology, being a well-differentiated NET with a much more favorable outcome. The age at presentation is 29 to 79 y/o, with an average of 45.3 years. The time of symptoms onset to diagnosis, also has a wide variability, from 5 days to 6 years, with no correlation between onset and grade of malignancy. In the literature is obvious that carcinomas have the worst prognosis, comparing them to well-differentiated, with overall survival of a few weeks and 7.5 years, respectively.

One feature that stands out is that 6 out of 11 primary NENs arise extra-axially. Two cases originated from the pineal parenchyma [5,8] and the other inside of the third ventricle [7]. On the other hand, when a NEN is metastatic, it usually metastasizes to the brain parenchyma [19], which is explained by the hematogenous dissemination. So, it seems that primary NENs are predominantly extra-axial lesions. The reason for this is not clear.

**Table 1:** Reported cases of primary brain neuroendocrine tumors in the world literature NS (not specified), CPA (cerebellopontine angle), ChT/RT (chemotherapy/radiotherapy)

Author (Reference)	Year	Onset to diagnosis	Sex	Age	Histology (Grade)	Localization	Treatment	Progression-Free Survival	Overall Survival (OS)
Porter et al [1]	2000	2 weeks	M	62	Low Grade	Right CPA	Subtotal resection	5 years	5 years
Deshaies et al [2]	2004	“months"	F	79	NS	Right Frontal Convexity	Total resection and octreotide	NS	6 weeks
Ibrahim et al [3]	2010	6 years	F	29	NS	Jugular foramen	Biopsy, and Somatostatin	1 year	1 year
Hood et al [4]	2014	4 months	F	61	Low Grade	Cavernous sinus	Subtotal resection	7.5 years	7.5 years
Tamura et al. [9]	2014	NS	M	77	High Grade	left temporal and parietal lobes	Total resection / RT	8.8 months	(1.9 years)
Hakar et al [5]	2016	NS	F	35	Intermediate Grade	Pineal Gland	Subtotal resection / ChT /RT	NS	26 months
Liu et al [6]	2016	2 months	F	39	High Grade	Sellar and Hypothalamus	Total resection and RT	NS	3 months
Liu et al [7]	2016	6 years	F	40	Low Grade	Anterior Skull Base	Total resection	NS	NS
Reed et al [8]	2019	NS	F	34	NS	Third ventricle	Total resection ChT / RT	10 years	10 years
Cheng et al [9]	2021	5 days	M	53	NS	Pineal gland	Partial resection and ChT	21 months	21 months
Stepien et al [10]	2022	NS	M	5	NS**	Left cerebellar	Total resection / RT	5 weeks	22 Months
Caro-Osorio et al (present case)	2022	6 months	M	29	High grade	Right CPA	Partial resection ChT/RT	8 weeks	3 months

## Conclusions

Primary brain NENs are rare lesions, particularly at the cerebellopontine angle. The first line of treatment for this type of tumor is surgery followed by radiation and chemotherapy. The prognosis of these tumors is correlated to their histological grade. The case we presented was a high-grade tumor with a limited prognosis.
